# A review of cost-effectiveness analysis: From theory to clinical practice

**DOI:** 10.1097/MD.0000000000035614

**Published:** 2023-10-20

**Authors:** Sara Michelly Gonçalves Brandão, Hans-Peter Brunner-La Rocca, Antonio Carlos Pedroso de Lima, Edimar Alcides Bocchi

**Affiliations:** a Instituto do Coracao do Hospital das Clinicas da Faculdade de Medicina da Universidade de Sao Paulo, Sao Paulo, Brazil; b Heart Failure Clinic, Department of Cardiology, Maastricht University Medical Center, Maastricht, Netherlands; c Institute of Mathematics and Statistics, Department of Statistics, Universidade de Sao Paulo, Sao Paulo, Brazil.

**Keywords:** bootstrapping and health policy, cost-effectiveness analysis, cost-utility analysis, health economics, QALY

## Abstract

Cost-effectiveness analysis has long been practiced; registries date back to the United States of America War Department in 1886. In addition, everyone does intuitive cost-effectiveness analyses in their daily lives. In routine medical care, health economic assessment becomes increasingly important due to progressively limited resources, rising demands, population increases, and continuous therapeutic innovations. The health economic assessment must analyze the outcomes and costs of actions and technologies as objectively as possible to guarantee efficient assessment of novel interventions for Public Health Policy. In other words, it is necessary to determine how much society or patients are willing to or able to pay for novel interventions compared with existing alternatives, given the available resources. In addition, increased cost may displace other health care services already provided in case of fixed budget health care systems. To conduct such analyses, researchers must use standard methodologies and interpretations in light of regional characteristics according to social and economic determinants as well as clinical practice. Such an approach may be essential for transforming the current healthcare system to a value-based model. In this narrative review, concepts of the importance of and some approaches to health economic evaluation in clinical practice will be discussed.

## 1. Introduction

The prevention and management of diseases require constant improvement that in general is based on innovation and new technology. The choice between new and existing interventions or programs to allocate resources presents important challenges associated with access and affordability that affect patients, policymakers, payers, and clinicians,^[1]^ even in developed countries.

In this setting, the cost-effectiveness analysis arises as a worthy technical tool for decision-making, not only for authorities of health care insurers, but also to help doctors in clinical practice. Contrary to what might be considered, it is not about giving monetary value to a life, but how much society, or patients, are willing to or able to pay for the efficacy of novel interventions compared with existing alternatives or if health care systems and patients may even save money with novel interventions. Better allocation of financial resources could be essential for individual patients and the whole population to prevent a “state of crisis” reported in expensive health care systems, which may even result in a reduction in deaths.^[2,3]^

Without technical and objective tools to assist doctors in the decision-making process, doctors may be faced with dilemma that is named popularly in some countries as Sophia choice.

Besides those, evaluating effectiveness in clinical practice without considering the balance between reduction in events and additional costs may be insufficient for the implementation of new interventions.^[5,6]^ Moreover, higher spending does not necessarily result in better outcomes. For instance, the health-adjusted life expectancy, which is the number of years in full health that an individual can expect to live given the current morbidity and mortality conditions, is lower in the United States compared with other high-income countries, although health spending in the United States is much higher.^[7]^

Although, cost-effectiveness concept in nonmedical issues dates back to the United States War Department in 1886^[8]^ and the origins of cost-effectiveness research in Western medicine predate to the 1930s,^[9]^ until now, the use of incremental cost-effectiveness ratios (ICER) in the decision-making process remains without consensus with different cost-effectiveness thresholds among regions.^[5,10,11]^

## 2. Health economic evaluation

Depending on available cost and effectiveness data, economic health assessment can be estimated in 2 ways. On the 1 hand, it may be based on actual (clinical) data, For example, from observational studies or clinical trials. On the other hand, it may be based on computerized modeling, using data that come from different sources, such as actual (clinical) data, systematic reviews, epidemiological studies, and expert opinions. In such analyses, data from different sources are often combined. Decision models are a schematic representation of the complexity of the real-world and demonstrate patients transition through different health stages.^[4,5,12]^

The economic evaluation studies conducted with a clinical trial, also called a piggyback study,^[13]^ have both advantages and disadvantages. Thus, they usually benefit from randomization and blinding, and it may be easier and cheaper to include an economic component within a prospective clinical trial than to finance an economic evaluation independently of a trial. However, the main disadvantages lie in the facts that clinical trials usually do not reflect “real-world” practice, and that the time horizon is limited to the time of follow-up of the study. In such cases, computer models can be used in a complementary way to estimate long-term effects or to apply the results to other patient populations with the same disease. Often, the modeling technique may be the only available approach to overcoming the above-mentioned shortcomings of clinical trials but has the limitation of being based on assumptions that cannot be tested as part of the trial.^[14,15]^

The main applied modeling techniques are the static models, such as decision trees and Markov modeling as well as dynamic models and microsimulation models.^[14–16]^ Obviously, it is necessary to evaluate the quality and appropriateness of such models regarding the available data used, the validity of models comparing the results obtained with other studies or models, the transparency by means of description and the justification for using certain models.^[16]^ However, the modeling techniques based on current assumptions may not be true in the future, because the area of health is constantly changing. Therefore, new technologies or products can add new results, altering the whole modeling scenario. The exact description of these modeling techniques are, however, beyond the scope of this review and are described in detail elsewhere.^[16]^

### 2.1. Types of methods of economic evaluation

Economic evaluation analyses (Table 1) are usually classified into 4 categories: cost-minimization, cost-effectiveness (CEA), cost-utility, and cost-benefit analysis.^[4]^ Details of these definitions are shown in (Appendix 1 Supplemental Digital Content, http://links.lww.com/MD/K290).

**Table 1:** Measures of health economic analyses: strengths and limitations.

Section/Item	Description	Strengths	Limitations
Types of studies economic evaluation			
Cost-minimization (CMA)	CMA measures and compares costs from equivalent outcomes	Simple method	Alternatives must have identical outcomes
Cost-effectiveness (CEA)	CEA measures costs in monetary unit and outcomes in natural units and is currently one of the most commonly used methods in economic evaluation.	Outcomes are reported as natural unit (e.g., life year [LY], blood pressure reduction, cardiovascular event avoided). LY is one of the most used methods because it allows different therapeutic interventions.	Most outcomes do not allow direct comparisons. For example, it is not possible to compare LY data of one study with another study with blood pressure reduction.
Cost-utility (CUA)	CUA costs are measured in monetary units, and outcomes in Quality Adjusted Life Years (QALY) or years of disability-adjusted life years (DALYs)	QALY is one of the most commonly used methods in economic evaluation, because it aggregates, for example, data of quality and quantity of life; besides this, it is possible to compare different interventions.	Diverse populations have different preferences and even specific time periods have differing treatment paradigms. Therefore, it can be difficult to make direct comparisons. QALY does not consider a variety of contextual factors (program-specificity, palliative care, mental health).
Cost-benefit (CBA)	CBA compares both costs and outcomes in monetary units.	CBA results can indicate intervention desirability independently of a comparison to alternatives (other economic evaluation methods cannot).	The practical difficulty of monetary valuation of benefits and the fundamental problem in health of placing a dollar value on human life (or other health outcomes) limit the use of CBA
Measurement of effectiveness			
Life years	LYs are calculated as the remaining life expectancy at the point of each averted death. Life expectancies may be taken from life tables that are specific for each setting or standardized across larger regions	LY is, as indicated, a relatively easy and transparent method for measuring population health.	The method ignores the obvious fact that health is more than merely staying alive. The method will fail to acknowledge health improvements, such as improved physical ability, reduced neuropsychological stress, and reduced chronic pain. LYs gained represent an intrinsic bias against conditions that are largely nonfatal.
QALY	Calculate the average utility of individual values between two consecutive time measurements and multiplying it by the time interval between the measurements.	Death is combined with morbidity by attaching a weight to each health state.	Insufficient sensitivity to measure small but clinically meaningful changes in health status, which is important to certain patient subgroups, for example, cancer patients, where multiple studies have outlined a need for additional dimensions to be considered.
DALY	Disability-adjusted life years (DALYs) are calculated by combining years of life lost (YLL) as well as the years lived with disability (YLD)	This consequently provided an objective and quantitative description of the gap between the ideal health status and actual population health status.	Difficult to measure, requires more work, is not very widespread.
Cost classification			
Direct medical costs	Expenses are directly linked to health professionals and treatment products	Relatively *easy* to measure	Expenses are often difficult to assess accurately
Nonmedical direct costs	Expenses of the patient and family are directly related to the treatment of the disease	Includes more information about the disease process	Difficult to estimate, requires more work, and these costs can vary substantially according to country or region.

### 2.2. Cost

Costs are calculated by identifying, quantifying, and valuing the different types of resources used. Costs can be classified as direct medical costs, nonmedical direct costs, indirect and intangible costs^[10]^ (Appendix 1 Supplemental Digital Content, http://links.lww.com/MD/K290).

To calculate direct medical costs, 2 methodologies are used, That is, micro-accounting or macro-accounting, or the combination thereof. Micro-costing represents a methodology in which each item of the resources used is estimated and a unit cost is attributed to it, resulting in cost estimates with a higher level of detail to identify the inputs consumed by focusing on the individual patient.^[17,18]^ In contrast, macro-costing consists of identifying the most relevant resources at a high level of aggregation, thus providing an average of treatment costs for each category of disease, for example, national registries such as Hospital Information Systems and the Ministry of Health’s Outpatient Information System apply macro-costing. The advantage is that it is usually a more feasible method than micro-costing. It presents, however, a lower degree of accuracy in cost estimates and may, therefore, be less sensitive for smaller differences in direct costs.^[17,18]^ On the other hand, micro-costing may neglect some costs that are assumed not to be influenced by the treatment or contribute only very little to the total cost to increase the feasibility of micro-costing.

The methods are further subdivided into bottom-up and top-down. In the top-down estimation, resources are valued from comprehensive sources, for example, the System of Management of the Table of Procedures, Medications, Orthotics and Prostheses, and Special Materials of the Unified Health System or Medicare’s Bundled. Alternatively, in the bottom-up approach, human, material, and financial resources are valued from hospital services, work, or purchase contracts.^[17]^

### 2.3. Perspective

Usually, the effective costs are not easily available and the perspective of how to evaluate costs differs between involved stakeholders. The definition of the perspective (point of view) of the economic analysis is fundamental for the identification of the costs to be considered. There are several perspectives, That is, the payer, the patient, the society. The methodological standards advocated by the Second Panel on Cost-Effectiveness recommend the perspective of society, because it incorporates all costs, That is, direct and indirect, regardless of who supports them. However, the decision makers may prefer other perspectives according to the specific need.^[19]^ From the perspective of the payer (Public Health System, health plan operator, private sector), the direct costs related to the intervention are measured, but the nonmedical direct cost or indirect cost are not considered because they are not financed by the payer.^[5,10,20]^

### 2.4. Adjustments of cost data

Cost and benefits occurring at different time points or incurred in different countries and currencies should be adjusted. In addition, cost collected at different time periods can be influenced by inflation. To remove the effects of inflation from analysis, it may be necessary to value all the resource on a common base year (usually the present) or adjust them. This adjustment can be done using the consumer price index or gross domestic price deflators.^[10,21]^

Also, discounting must be used to convert the value of benefits and costs that will occur in the future at different timepoints. It is necessary to adjust for this when the project works with projection, for example in a Markov model. The discount rate varies according to each country and remains a matter of debate in the literature.^[22]^ The National Institute for Health and Clinical Excellence in England uses equal tax discounting at 3.5%, but there is considerable variation among European countries, with discount rates varying between 3 and 5%.^[22]^ In Brazil, the discounting rate recommended is 5%,^[1]^ whereas the United States Federal Government mandates using a discount rate of 3%.^[23]^

### 2.5. Converting cost into a common currency

Furthermore, a simple translation of all national unit cost into 1 common currency using market exchange rates from currency markets does not reflect the different price levels between countries. If there is the intention-to do so, economists often opt for a hypothetical currency, called “international dollars”. The idea is that a given amount of international dollars should buy roughly the same amount – and quality – of goods and services in any country.^[5,21,24]^

“Purchasing power parities are the rates of currency conversion that equalize the purchasing power of different currencies by eliminating the differences in price levels between countries.”^[24]^

### 2.6. Effectiveness: Calculating quality adjusted life years (QALY)

“QALY can be obtained manually by calculating the average utility of individual values between 2 consecutive time measurements and multiplying it by the time interval between the measurements and summing up all the values.”^[25,26]^ The average QALY of a group can be estimated for each time period and/or the sum of all periods together (if all patients have the same chance to complete the periods). Estimation of mean QALY is complex given that some individual have survival time censored. Therefore, when computing QALY, such censored observations will turn out to be informative, making the usual Kaplan–Meier estimation inadequate. In such a case, an alternative is to consider the other method.^[27]^ The same authors developed a method to estimate the mean QALY.^[28]^ Hence, differences in QALY between groups can be analyzed by looking into differences in the corresponding means.

Patients with baseline utility data who died before the first follow-up utility assessment should be included, with the area under the curve estimated based on time intervals from baseline utility data collection to death. Balancing QALY may be necessary.^[29,30]^ (Appendix 1 Supplemental Digital Content, http://links.lww.com/MD/K290).

### 2.7. Sensitivity analysis

Sensitivity analysis is a powerful tool for examining the impact of data uncertainty due to different study methodologies, data sources, and lack of or because of data, among others.^[5,19]^ Its purpose is to ascertain the robustness of the results by changing different variables to determine how much the variation can influence the final result. Sensitivity analysis also allows exploring the generalization of results.^[31]^ Variations in data should be justified by literature review, expert consultation, or using confidence intervals.^[5,19,31]^ Sensitivity analyses can be deterministic or probabilistic.^[5,32,33]^

More elaborated and highly recommended probabilistic sensitivity analysis (PSA)^[5,19,31]^ conducts simulation by means of the Markov Chain, Monte Carlo simulation, or bootstrapping.^[10,34,35]^ PSA can be conducted both in economic evaluation alongside a clinical trial as well as in a decision analytic model. PSA has become the standard because it permits the joint uncertainty across all parameters in the model to be assessed at the same time. We will describe in detail below the bootstrap approach, which is our focus.

#### 2.7.1. Bootstrap method.

Because incremental ICER and incremental cost-utility ratio (ICUR) are fractions and the underlying variables are usually not normally distributed, it is inappropriate to use standard statistical techniques to construct confidence intervals. One possible solution is to consider other techniques such as nonparametric bootstrapping.^[36]^

The bootstrap method is a statistical technique where random samples of equal size are drawn, which are used as the original sample. Each bootstrap sample may contain some of the original observations more than once, whereas other components of the original sample may not be chosen. There is no definition as to the number of samples, but at least 1000 is recommended.^[36]^ The basic concept behind the bootstrap is to treat the study sample as if it were a conceptual population. It is better to draw inferences from these samples than to make potentially unrealistic assumptions about the underlying population. Bootstrapping provides insight into the distribution of results and the accuracy of the estimation.

A series of procedures was developed for constructing bootstrap confidence intervals, which include a normal approximation method, a percentile method, the *t* percentile method, the bias-corrected percentile, and the accelerated method of bias correction. The ideal choice among those methods is, however, specific for the application at hand. Several authors provide a complete description of each technique along with a summary of the advantages and disadvantages of each 1.^[36–39]^ A complete discussion of all these techniques is beyond the scope of this article.

One of the main advantages of the bootstrap method is that one does not have to make use of distributional assumptions for the data, even when 1 relies on asymptotic results (i.e., large sample results) to draw conclusions. In fact, the method may be applied to any estimator, even complicated ones like ICER or ICUR.

In some situations where bootstrap is used to construct confidence intervals, the actual confidence level may be different from the desired 1, and more sophisticated methods need to be incorporated. In addition, for small sample sizes the method may not be applicable. Most of the time, cost and outcome data are not normally distributed, consequently there is a tendency to employ media values, because they are greatly influenced by extreme values.^[21]^

If number of samplings is high, which is recommended, averages are (almost) equal to the results based on parametric tests. The value of bootstrap is therefore primarily to get insight into the distribution of data and the likelihood of reaching certain thresholds (Appendix 1 Supplemental Digital Content, http://links.lww.com/MD/K290).

### 2.8. Incremental cost-effectiveness planes

**Figure 1.**
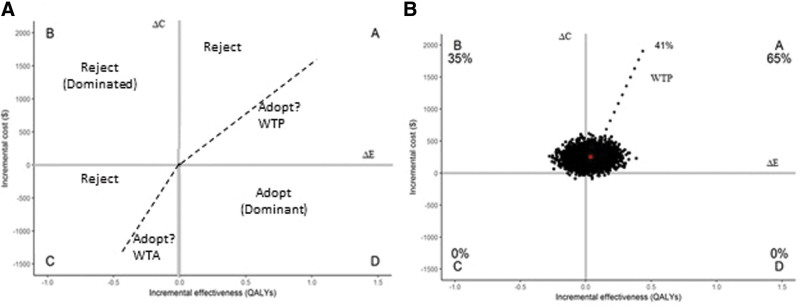
Cost-effectiveness planes. Δ*C* and Δ*E* = differences in cost and effectiveness among health interventions, respectively, GDP = gross domestic product, WTA = willingness to accept, WTP = willingness to pay threshold, $ = American dollar. The red circle represents the estimated mean values of our sample. “A,” “B,” “C,” and “D” are the quadrants of the scatter plot. Percentages give frequencies of samples in each quadrant. Dashed line represents 0.5 GDP per capita, $4381. Source: A, created by the author; B, from Brandao et al^[45]^

**Figure 2.**
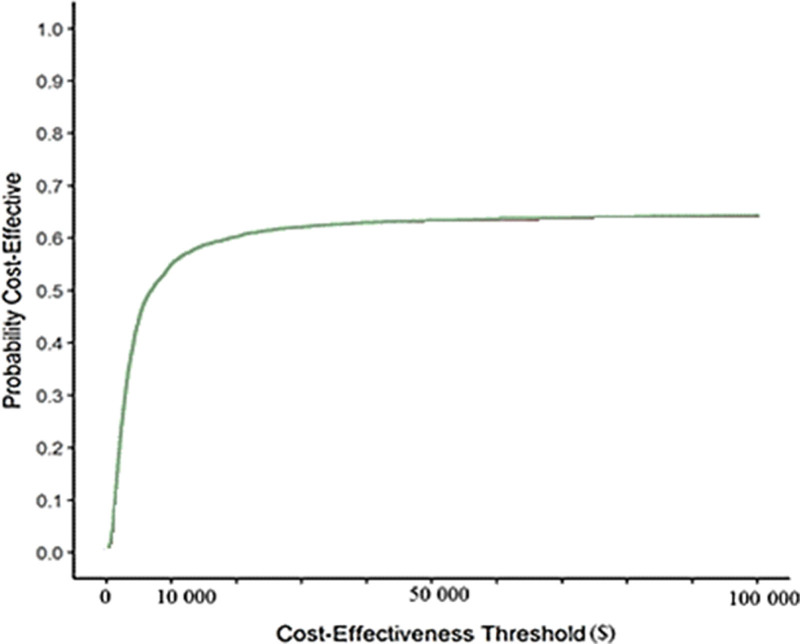
Cost-effectiveness acceptability curve. Cost-effectiveness acceptability curves for quality adjusted life-years. The *x*-axis represents the willingness to pay by society in dollars ($). The *y*-axis represents the probability of therapy A being cost-effectiveness compared with therapy B. Source: Brandao et al^[45]^

Cost-effectiveness analysis using the nonparametric bootstrap technique can be represented graphically in the form of incremental cost-effectiveness planes by means of a scatter plot (Fig. 1) and/or cost-effectiveness acceptability curve (CEAC) (Fig. 2).^[10,34,35]^

Incremental cost-effectiveness planes are used to plot the incremental costs and incremental effectiveness of an alternative therapy relative to the comparator and to indicate the uncertainty about the alternative therapy being cost-effective or not. The incremental effectiveness is presented on the x-axis, whereas the incremental cost is presented on the y-axis. Each of the samples of bootstrapping represent 1 point of the scatterplot in 4 quadrants described below. The 2.5 and 97.5 percentiles calculated in Figure 1 represent the 95% confidence interval of the ICER or ICUR.^[10]^

Quadrant A (North East): Alternative treatment proves to be more effective and more costly. Thus, a greater benefit can be obtained at an additional cost (Fig. 1A).

Quadrant B (North West): Alternative treatment is more costly and less effective than the standard. This scenario is termed “Dominated” and is obviously always rejected (Fig. 1A).

Quadrant C (South West): Alternative treatment proves to be less effective and less costly. Economy can be achieved but is associated with a loss of effectiveness. Usually, such a scenario is considered not acceptable even if the cost savings are relatively large (Fig. 1A).

Quadrant D (South East): Alternative treatment is more effective and less costly, termed “Dominant.” Such a scenario is always accepted (Fig. 1A).

Decision-making may depend on the density present in the quadrants and the willingness to pay (WTP). Thus, if much of the density present in the quadrants is below the WTP (i.e., results in quadrant A below the WTP threshold), treatment may be accepted by the decision maker (Fig. 1B). However, if the bulk of the density is above the WTP, the treatment should be rejected. CEAC are used to summarize the uncertainty of the Incremental cost-effectiveness.

### 2.9. Cost-effectiveness acceptability curve

The CEAC estimates the likelihood that the proposed therapy is cost-effective compared with the standard treatment at different levels of WTP.^[40]^

The x-axis presents different levels of WTP and the y-axis provides the probability of the alternative intervention being cost-effective at a range of thresholds of WTP. Thus, if the cost-effectiveness threshold is changed, the probability of cost-effectiveness also changes.^[40]^

The CEAC construction (Fig. 2) involves the density of the quadrants in the dispersion graphs that are below the maximum limit to be paid.^[40]^ Thus; If the alternative intervention falls into the B and C quadrants (i.e., is less effective), CEAC will not reach 1 on the Y-axis at infinitively high WTP; If there is density in the C and/or D quadrants, the CEAC will intersect the Y-axis in the percentile corresponding to this density; The CEAC does not constitute the cumulative density of the quadrants of the scatter plot.^[40]^ Sometimes, a less effective alternative intervention is considered not acceptable even if it is cost-saving. In this case, the C quadrant may be equally treated as the B quadrant.

### 2.10. The influence of risk/events and the accuracy of studies in CEA

The ICER/ICUR results may be influenced by the event rates in each population. For instance, a recent study in patients with a very high risk of atherosclerotic cardiovascular disease showed that ICERs range from $59,331 to $10,584, depending on the varying level of risk within the population at very high risk. In the analysis in which the event rate of 12.4 events per 100 patient-years was considered, the ICER was $10,584. On the other hand, the ICER for 6.4 events per 100 patient-years was $59,331.^[41]^ Accordingly, the extrapolation of ICER results for population with different risk and event rates is of particular concern. For instance, heart failure cost-effectiveness results based on patients in New York heart association class II or III cannot be directly extrapolated to New York heart association class I because the event rate is lower in comparison with that in class II-III.^[42]^

Also, data that will be used for cost-effectiveness should be derived from robust trials. Characteristics of design and the results of interventions should be analyzed carefully. Special attention should be paid to; Bias by excluding patients who had adverse events of the intervention threatening the concept of intention-to-treat and creating/expanding positive results; The lack of sham in non-blinded interventions; characteristics of included patients that differ from the inclusion criteria (e.g., not all functional classes were included or the distribution is different than anticipated); Replacing or using of new therapy while standard therapy was not yet optimized according to the guidelines; The percentage of missing patients during follow-up; Quality according to the CONSORT criteria.^[43]^ Effects on surrogate endpoints should be interpreted carefully considering the limitations.

Recently, the fragility index was proposed to help to interpret the robustness of the results of randomized clinical trials.^[44]^ The fragility index is the minimum number of patients whose event status would change the statistical significance result; the smaller the Fragility Index, the more fragile the trial’s. The smaller the fragility index, the more fragile the trial results are. Importantly, the number of patients lost to follow-up in the treatment group should always be lower than the fragility index.

In addition, there are concerns with the potential budget impact of the introduction of new and more costly drugs added simultaneously to current medicine for 1 disease mainly if they were not tested together or if there is no longer the opportunity to test them together.

### 2.11. Approach to choosing interventions in clinical practice considering CEA

*“The right drug for the right patient at affordable cost”.*

**Figure 3.**
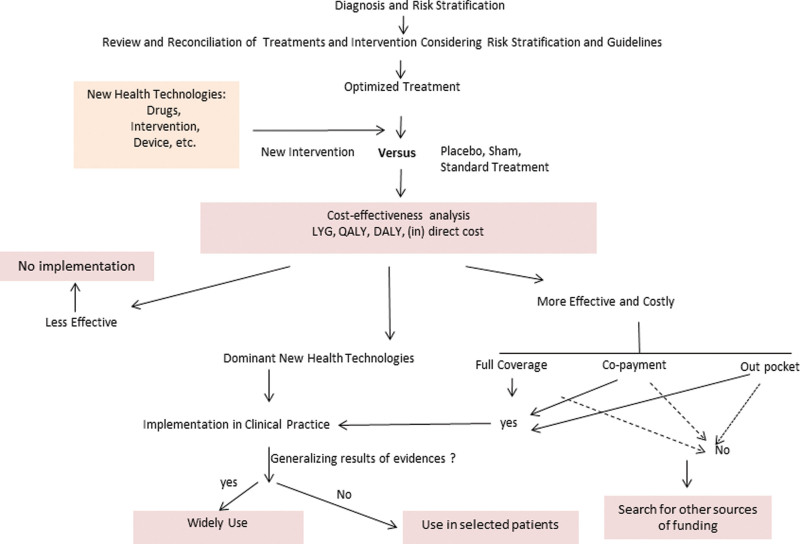
Flowchart for cost-effectiveness in implementation of new health technologies on top of optimized treatment, according to guidelines or replacing other treatment in clinical practice. DALY = disability adjusted life year, LYG = life-years gained, QALY = Quality Adjusted Life Years. Source: created by the author.

In clinical practice, selecting patients for the right individualized treatment using CEA is a challenge. Figure 3 illustrates a suggestion of how the clinical decision-making process may consider CEA.

### 2.12. Limitations of CEA

According to some authors, a delimited ICER threshold does not take into account the fact that decision makers can choose to apply a varying ICER limit, according to the type of medicine, the type of disease and the decision-making context.^[34,35]^ In fact, the variable threshold ICER model is applied in some countries. It is believed that the ICER threshold should be greater for drugs due to the higher social value or for rare diseases for which there is no alternative therapy. Also, thresholds for ICER are defined arbitrarily.^[35]^

Furthermore, clinical trials often include a highly selected population for a certain period, largely neglecting an appropriate lifetime horizon. Consequently, the results may not reflect all patients with a certain disease in the real-world setting, or might be considered insufficient to evaluate events.^[10]^

Moreover, projections generally assume that the patients’ characteristics, dosages, and surrogate measures are homogeneously distributed over time, but often this is not the case. In addition, data are often missing. Assumptions may be necessary but may not cover the full clinical complexity. In addition, long-term projections need more data to support them, and that may not always be available, particularly for new therapies, and extrapolating data can introduce bias.

## 3. Conclusion

CEA is increasingly important for decision-making in health care and likely in future clinical practice. Because resources are limited, it may be useful not only for the formulation of public policies, but effort should be made for CEA to be considered also by health care professionals in the daily clinical decision-making process. Shared decision-making would be encouraged in clinical practice if patients are adequately informed about efficacy and CEA of any therapeutic option, particularly if it includes out-of-pocket expenses.

## Author contributions

**Conceptualization:** Sara Michelly Gonçalves Brandão.

**Formal analysis:** Sara Michelly Gonçalves Brandão.

**Methodology:** Sara Michelly Gonçalves Brandão.

**Writing – original draft:** Sara Michelly Gonçalves Brandão, Edimar Alcides Bocchi.

**Writing – review & editing:** Sara Michelly Gonçalves Brandão, Hans-Peter Brunner-La Rocca, Antonio Carlos Pedroso de Lima, Edimar Alcides Bocchi.

## Supplementary Material


