# Supplementary material for: A review of cost-effectiveness analysis: From theory to clinical practice
Source: Medicine (Baltimore). 2023 Oct 20;102(42):e35614. doi: 10.1097/MD.0000000000035614 (PMC10589545; doi:10.1097/MD.0000000000035614)
Supplement: Supplementary file 1 [file medi-102-e35614-s001.docx]

**Appendix" to "Supplemental Digital Content (Appendix)**

**A REVIEW OF COST-EFFECTIVENESS ANALYSIS: FROM THEORY TO CLINICAL PRACTICE**

**Types of methods of economic evaluation**

Cost-minimization (CMA) measures and compares costs from different interventions with identical outcomes. In principle, it is a relatively simple method because only costs have to be considered. However, its use is limited because alternatives must have identical outcomes, which is often not the case, is difficult to prove, or is subject to uncertainty. Cost-benefit analysis (CBA) compares both costs and outcomes in monetary units. CBA is not widely used by health professionals, because it attributes economic values to health outcomes, which is seen as questionable from the clinical point of view.^(1-3)^ Therefore, only cost-effectiveness (CEA) and cost-utility (CUA) are discussed in more detail.

Cost-effectiveness analysis

CEA measures costs in monetary units and outcomes in clinical events. It is currently one of the most commonly used methods in economic evaluation. Outcomes are assessed as events, including years of life (LY), avoided events (eg, hospitalizations or invasive interventions), or reductions in laboratory and clinical measures. Also, combinations of these outcomes can be used (eg, years outside of hospital; combined events, such as mortality, hospitalizations, and interventions), which have the limitation of possibly being difficult to directly compare different measures of clinical outcomes. Among the outcomes, LY is the most used method. The endpoint all-cause mortality is scientifically the most rigorous endpoint.^(4)^ It is measurable, robust, with no bias and has minimal confounding factors. However, it ignores improvements in other important clinical factors, such as pain, physical capacity, other symptoms or quality of life.^(5, 6)^ Thus, the most important disadvantage is that the method ignores the obvious fact that health is more than merely staying alive or being without an event.

The incremental cost-effectiveness ratio (ICER) is a result of the differences in costs (C) divided by differences in outcomes (E) from two independent samples or intervention groups and is presented as: ICER = (CA- CB)/(EA- EB) where CA and CB, respectively, are the average cost of treatment or group A (alternative) and treatment or group B (standard), and EA and EB represent the mean effectiveness in group A and B. For example, effectiveness is the mean LY in each group or the mean number of events.

Cost-utility analysis

In CUA, costs are measured in monetary units, and outcomes consider a combination of survival time and quality of life, usually expressed as Quality Adjusted Life Years (QALY) or Disability-Adjusted Life Years (DALYs).^(1-3)^ These measures capture simultaneous gains from survival and quality of life. As CUA supports the concept that the therapeutic goal should not be limited to prolonging survival but that improving quality of life is equally important, it is currently the preferred method of most analysts.^(5)^ However, some authors do not distinguish between CEA and CUA.^(3)^

QALY integrates indicators of quantity (mortality) and health-related quality of life (HRQL; morbidity) as described below, whereas the calculation of the DALY is quite complex.^(5)^ The incremental cost-utility ratio (ICUR) is presented as: ICUR = (CA- CB)/(QALYA- QALYB) where CA and CB, respectively, are the average costs of treatment or group A (alternative) and treatment or group B (standard), whereas QALYA and QALYB represent the mean QALYs of treatment/group A and treatment/group B.

**Cost classification**

Costs can be classified as direct medical costs, nonmedical direct costs, indirect and intangible costs.

Direct medical costs involve expenses directly linked to health professionals and treatment products. In contrast, nonmedical direct costs are the expenses of the patient and family that are directly related to the treatment of the disease. This may include, for example, the transport of the patient to the health care facility, the cost of lodging the family when the hospital is situated in another city, or costs related to food. Indirect costs are those resulting from the loss of productivity of the patient or caregiver involved in the treatment. In turn, the intangible costs are those related to the suffering of the patient and family, to pain, tiredness, depression, social exclusion, among others. It is difficult to exactly express these sufferings in a monetary value.^(5)^

**Transforming quality of life in utilities and nationally representative preferences**

Utilities can be obtained from a generic questionnaire on quality of life. For example, the items in the Short Form Health Survey 36 (SF-36) can be converted into a 6-dimensional health state classification system to obtain utilities based on the Short-Form 6 dimensions (SF-6D). The SF-6D is a single-index summary preference-based measure of health derived from 11 items of the SF-36, allowing for a total of 18,000 distinct health states.^(7)^

It is important to point out that the Second Panel on Cost-Effectiveness in Health and Medicine (SPCEHM) recommends the use of community-based nationally representative preferences for health states for use in CEA.^(8)^ In the case of Brazil, for instance, the SF-6D algorithm generates health state values using a representative and validated sample of the Brazilian general population from the capital city of Rio Grande do Sul ^(9)^ to approximate the societal viewpoint.

**Utility**

HRQL can be evaluated in two ways. First, it can be measured by means of psychometric measures, such as the generic Medical Outcomes Study SF-36. Psychometric measures are not widely used in health economics, although utility indexes can be derived by a quality of life questionnaire.^(7)^ Consequently, they do not allow comparisons between different interventions.^(1, 10, 11)^ Second, HRQL can be derived directly from preferences or utilities, based on the value that society attributes to a certain state of health.^(5)^

The utility is usually expressed on a numerical scale ranging from 0 to 1, which represents death and perfect health, respectively. Utilities can be estimated by various techniques, through interviewing applications for direct utility measures or indirect health utility measures. In the first method, individual preferences and perceptions are captured by for example, the Visual Analog Scale, Standard Gamble, or Time Trade-Off. In the second, utilities are estimated using questionnaires, such as Health Utility Index (HUI), SF-6D, Euroqol-5D (EQ-5D),^(1, 2)^ which are among the most used instruments internationally to measure health status preferences and to generate QALYs.(7) The scores are based on measured preferences in the general population, allowing the calculation of utility scores and the comparisons between different interventions and different patient profiles.

**Balancing QALY**

A tool to reduce heterogeneity is to perform large randomized controlled trials; in spite of this, imbalance may remain.^(12)^ Estimating QALYs when the groups have different baseline utility will usually bias the result because those who start out with higher utility accrue the most QALYs.(13) Therefore, additional regression analyses are needed to explore to what extent baseline differences explain later differences.^(12)^

**Deterministic and Probabilistic analyses**

Deterministic analyses can be univariable, multivariable, or performed as various scenarios. Probabilistic analyses are defined based on probability distribution functions, where selected parameters vary according to these distributions.^(1, 14, 15)^ In the univariate analyses, each variable can be represented graphically through a tornado diagram, using a fixed range of the parameter. Another way to preset univariate analyses is the threshold analysis where the value is calculated at which the (novel) intervention becomes cost-effectiveness. In the multivariate analyses, two or more base case variables are modified simultaneously. In scenario analyses, a set of optimistic, pessimistic, or more and less likely scenarios are created. One or more variables can be considered and the variation of their parameters is done by determining their minimum and maximum values.

**Bootstrap method**

A series of procedures was developed for constructing bootstrap confidence intervals, which include a normal approximation method, a percentile method, the *t*-percentile method, the bias-corrected percentile, and the accelerated method of bias correction. The ideal choice among those methods is, however, specific for the application at hand. Several authors provide a complete description of each technique along with a summary of the advantages and disadvantages of each one.^(16-19)^ A complete discussion of all these techniques is beyond the scope of this article.

One of the main advantages of the bootstrap method is that one does not have to make use of distributional assumptions for the data, even when one relies on asymptotic results (ie, large sample results) to draw conclusions. In fact, the method may be applied to any estimator, even complicated ones like ICER or ICUR.

In some situations where bootstrap is used to construct confidence intervals, the actual confidence level may be different from the desired one, and more sophisticated methods need to be incorporated. In addition, for small sample sizes the method may not be applicable. Most of the time, cost and outcome data are not normally distributed, consequently there is a tendency to employ media values, because they are greatly influenced by extreme values.^(20)^

If number of sampling is high, which is recommended, averages are (almost) equal to the results based on parametric tests. The value of bootstrap is therefore primarily to get insight into the distribution of data and the likelihood of reaching certain thresholds.

**Cost-effectiveness and cost-utility threshold**

The willingness to pay (WTP) threshold represents the maximum per unit of outcome gained (QALY, LY, DALY) that the health care provider is willing to pay for the alternative therapy. Many countries have formal requirements for the submission of cost-effectiveness data.

Establishing cost-effectiveness thresholds is arbitrary ^(21, 22)^ and based on (1) demand values focused on the preferences of the affected population or (2) supply values focused on the opportunity cost of the intervention, ie, the cost per unit of effect of the most beneficial alternative that would not be funded if the intervention were implemented.^(23)^ Currently, in the UK the NICE uses a threshold of £30,000 per QALY, although this threshold has been considered too high by health economists at the University of York.^(24)^

On the other hand, some countries, for instance Brazil, do not yet have an explicit threshold defined.^(1)^ So these countries use the most widely cited cost-effectiveness threshold recommended by the World Health Organization (WHO) based on the country’s per capita gross domestic product (GDP), although the WHO has recently discouraged this recommendation.^(25)^ Also, it is important to point out that the effectiveness denominators (QALY, LY, DALY) are also used interchangeably without the necessary adjustments. Most studies use in the denominator QALYs or LYs gained rather than DALYs avoided, proposed initially by the WHO.^(26)^

**SUPPLEMENTAL REFERENCES**

1. Brasil. Ministério da Saúde. Secretaria de Ciência, Tecnologia e Insumos Estratégicos. Diretrizes metodológicas : Diretriz de Avaliação Econômica. Brasília: Ministério da Saúde; 2014. Available from: <http://bvsms.saude.gov.br/bvs/publicacoes/diretrizes_metodologicas_diretriz_avaliacao_economica.pdf>.

2. Neumann PJ, Goldie SJ, Weinstein MC. Preference-based measures in economic evaluation in health care. Annu Rev Public Health. 2000;21:587-611. PubMed PMID: 10884966. eng.

3. Gold MR, Siege JE, Russell LB, Weinstein MC. Cost-Effectiveness in Health and Medicine 1996. Available from: <https://www.oxfordscholarship.com/view/10.1093/acprof:oso/9780190492939.001.0001/acprof-9780190492939>.

4. Zannad F, Garcia AA, Anker SD, Armstrong PW, Calvo G, Cleland JG, et al. Clinical outcome endpoints in heart failure trials: a European Society of Cardiology Heart Failure Association consensus document. Eur J Heart Fail. 2013 Oct;15(10):1082-94. PubMed PMID: 23787718. Epub 2013/06/22. eng.

5. Drummond MF, Sculpher MJ, Claxton K, Stoddart GL, Torrance GW. Methods for the economic evaluation of health care programmes, 3rd ed. Thrid ed. New York: OUP Oxford; 2006. p. 464.

6. Willan AR, Briggs AH. Statistical Analysis of Cost‐effectiveness Data. First ed2006.

7. Brazier J, Roberts J, Deverill M. The estimation of a preference-based measure of health from the SF-36. J Health Econ. 2002 Mar;21(2):271-92. PubMed PMID: 11939242. Epub 2002/04/10. eng.

8. Sanders GD, Neumann PJ, Basu A, Brock DW, Feeny D, Krahn M, et al. Recommendations for Conduct, Methodological Practices, and Reporting of Cost-effectiveness Analyses: Second Panel on Cost-Effectiveness in Health and Medicine. Jama. 2016 Sep 13;316(10):1093-103. PubMed PMID: 27623463. Epub 2016/09/14. eng.

9. Cruz LN, Camey SA, Hoffmann JF, Rowen D, Brazier JE, Fleck MP, et al. Estimating the SF-6D value set for a population-based sample of Brazilians. Value Health. 2011 Jul-Aug;14(5 Suppl 1):S108-14. PubMed PMID: 21839880. Epub 2011/08/24. eng.

10. National Institute for Health and Clinical Excellence. Guide to the methods of technology appraisal 2013 2013 [Available from: <https://www.nice.org.uk/process/pmg9/resources/guide-to-the-methods-of-technology-appraisal-2013-pdf-2007975843781>.

11. Campolina AG, Bortoluzzo AB, Ferraz MB, Ciconelli RM. Validation of the Brazilian version of the generic six-dimensional short form quality of life questionnaire (SF-6D Brazil). Cien Saude Colet. 2011 Jul;16(7):3103-10. PubMed PMID: 21808898. Epub 2011/08/03. Validacao da versao brasileira do questionario generico de qualidade de vida short-form 6 dimensions (SF-6D Brasil). por.

12. Manca A, Hawkins N, Sculpher MJ. Estimating mean QALYs in trial-based cost-effectiveness analysis: the importance of controlling for baseline utility. Health Econ. 2005 May;14(5):487-96. PubMed PMID: 15497198. Epub 2004/10/22. eng.

13. Richardson G, Manca A. Calculation of quality adjusted life years in the published literature: a review of methodology and transparency. Health Econ. 2004 Dec;13(12):1203-10. PubMed PMID: 15386669. Epub 2004/09/24. eng.

14. Briggs A, Sculpher M, Buxton M. Uncertainty in the economic evaluation of health care technologies: the role of sensitivity analysis. Health Econ. 1994 Mar-Apr;3(2):95-104. PubMed PMID: 8044216. Epub 1994/03/01. eng.

15. Briggs AH, Mooney CZ, Wonderling DE. Constructing confidence intervals for cost-effectiveness ratios: an evaluation of parametric and non-parametric techniques using Monte Carlo simulation. Stat Med. 1999 Dec 15;18(23):3245-62. PubMed PMID: 10602149. Epub 1999/12/22. eng.

16. Briggs AH, Wonderling DE, Mooney CZ. Pulling cost-effectiveness analysis up by its bootstraps: a non-parametric approach to confidence interval estimation. Health Econ. 1997 Jul-Aug;6(4):327-40. PubMed PMID: 9285227. Epub 1997/07/01. eng.

17. Wakker P, Klaassen MP. Confidence intervals for cost/effectiveness ratios. Health Econ. 1995 Sep-Oct;4(5):373-81. PubMed PMID: 8563835. Epub 1995/09/01. eng.

18. Drummond MF. Confidence intervals for cost/effectiveness ratios. Publications OM, editor1980. 148 p.

19. Mooney CZ, Duval RD. Bootstrapping: a non-parametric approach to

statistical inference. First ed. Newbury Park CA, Sage1993. 80 p.

20. Gray AM, Clarke PM, Wolstenholme JL. Applied Methods of Cost-effectiveness Analysis in Healthcare. First ed. United States of America: Oxford University Press; 2010.

21. Marseille E, Larson B, Kazi DS, Kahn JG, Rosen S. Thresholds for the cost-effectiveness of interventions: alternative approaches. Bull World Health Organ. 2015 Feb 1;93(2):118-24. PubMed PMID: 25883405. PMCID: PMC4339959. Epub 2015/04/18. eng.

22. Pandey H, Paulden M, McCabe C. Theoretical models of the cost-effectiveness threshold, value

assessment, and health care system sustainability: Institute of Health

Economics; 2018.

23. Robinson LA, Hammitt JK, Chang AY, Resch S. Understanding and improving the one and three times GDP per capita cost-effectiveness thresholds. Health Policy Plan. 2017 Feb;32(1):141-5. PubMed PMID: 27452949. Epub 2016/07/28. eng.

24. Claxton K, Martin S, Soares M, Rice N, Spackman E, Hinde S, et al. Methods for the estimation of the NICE cost effectiveness threshold. Revised Report Following Referees Comments 2015 [Available from: <http://www.journalslibrary.nihr.ac.uk/hta>.

25. Bertram MY, Lauer JA, De Joncheere K, Edejer T, Hutubessy R, Kieny MP, et al. Cost-effectiveness thresholds: pros and cons. Bull World Health Organ. 2016 Dec 1;94(12):925-30. PubMed PMID: 27994285. PMCID: PMC5153921. Epub 2016/12/21. eng.

26. Soarez PC, Novaes HMD. Cost-effectiveness thresholds and the Brazilian Unified National Health System. Cad Saude Publica. 2017 May 18;33(4):e00040717. PubMed PMID: 28538787. Epub 2017/05/26. Limiares de custo-efetividade e o Sistema Unico de Saude. eng

por.
